# An explainable machine learning pipeline for prediction of antimicrobial resistance in *Pseudomonas aeruginosa*

**DOI:** 10.1093/bioadv/vbaf190

**Published:** 2025-08-22

**Authors:** Aakriti Jain, Govinda Rao Dabburu, Bishal Samanta, Neelja Singhal, Manish Kumar

**Affiliations:** Department of Biophysics, University of Delhi, New Delhi, Delhi, 110021, India; Department of Biophysics, University of Delhi, New Delhi, Delhi, 110021, India; Department of Biotechnology, National Institute of Technology Durgapur, Durgapur, West Bengal, 713209, India; Department of Biophysics, University of Delhi, New Delhi, Delhi, 110021, India; Department of Biophysics, University of Delhi, New Delhi, Delhi, 110021, India

## Abstract

**Motivation:**

Prediction of antimicrobial resistance in *Pseudomonas aeruginosa* using machine learning and genomic sequences holds the potential to serve as comparable alternatives to laboratory based detection if not better. Additionally, model interpretability can further enhance the potential of these models paving way for their reproducibility.

**Results:**

We have developed a machine-learning based 2-tier pipeline to predict resistance phenotype in *P. aeruginosa* using only genomic sequences as input in the form of k-mers. Our Decision Tree Model yields an accuracy of 79% and area under the receiver operating characteristic curve of 0.77 with a 70% specificity and 84% sensitivity. We have interpreted the model’s predictions using explainable AI as an attempt to bridge the gap between computational prediction and biological insight. Through these interpretations we have gathered antibiotic specific k-mer signatures pushing phenotype towards resistance.

**Availability and implementation:**

The curated dataset and related codes are available on request.

## 1 Introduction

*Pseudomonas aeruginosa* is a gram-negative bacterium which can infect all eukaryotes, including higher vertebrates and plants. For humans, *P. aeruginosa* is an opportunistic pathogen that infects primarily hospitalized and immunocompromised patients (Mathee *et al.* 2008, Jeukens *et al.* 2019, Spagnolo *et al.* 2021). Recent studies have reported that *P. aeruginosa* is the most common cause of nosocomial pneumonia and other hospital acquired infections like, urinary tract or surgical-site infections resulting in a significant number of deaths (Litwin *et al.* 2021). In the WHO Bacterial Priority Pathogens List, 2024, *P. aeruginosa* is kept under the “High Group” (World Health Organization 2024). Drug resistant bacteria under this group are significantly difficult to treat, cause a substantial disease burden (mortality and morbidity), show increasing trends in resistance, are difficult to prevent, are highly transmissible and there are few potential treatments in the development pipeline. Although they may not be critical globally, pathogens in this category could be critical for some populations and in specific geographical areas (World Health Organization 2024).

Conventional bacterial antibiotic susceptibility testing methods (AST) are the most popular methods to determine the antibiotic susceptibility of a bacterial pathogen. AST is the gold-standard method of antibiotic resistance profiling. However, an AST typically requires 48-72 hours to complete because it involves culturing bacteria and detecting drug resistance by either the disc-diffusion method or by conducting an assay to probe the presence of a specific chemical compound (Benkova *et al.* 2020, Gajic *et al.* 2022). On the other hand, molecular diagnostic methods like PCR are rapid and sensitive but cannot provide reliable results independently due to their limited specificity (Kurkela and Brown 2009, Pan *et al.* 2022, Wang *et al.* 2022, Liu *et al.* 2023). These methods may lack specificity as they often target genes or specific genetic markers only, requiring additional tests for accurate results (Yamin *et al.* 2023). Thus, these are not feasible for rapid testing on a mass scale and there is a pressing need to develop innovative and accurate solutions to discern the antimicrobial resistance (AMR) profile of a pathogenic microbe (Gajic *et al.* 2022). Deciphering the AMR profile of a bacterium using the traditional sequence alignment-based approaches is limited to known data. Hence, whole genome sequencing-based methods can be an alternative approach for predicting the resistance phenotype of a bacterial pathogen (Gupta and Verma 2019, Köser *et al.* 2014, Vanstokstraeten *et al.* 2023). Whole genome-based methods have also become promising alternatives due to a continuous decline in genome sequencing costs. Both factors have resulted in the availability of a large volume of genomic data.

The abundant availability of whole genome sequences offers a promising approach for resistance phenotype prediction through machine-learning methods that can be used as an alternative to the traditional sequence alignment-based methods. Prediction of antibiotic resistance profile using genomic sequences and machine-learning has been successfully implemented in many bacterial pathogens like *Escherichia coli* and *Klebsiella pneumoniae* (Sakagianni *et al.* 2023).

In this study, we have described a comprehensive pipeline for the prediction of antibiotic resistance in *P. aeruginosa* using a two tier machine learning framework based on k-mer analysis. In the first tier, the model predicts resistant or sensitive phenotypes of *P. aeruginosa* and in the second tier it predicts the specific antibiotic against which resistance has been reported. We evaluated various machine learning algorithms and the results of the best model were further interpreted through explainable AI techniques. The model has also been validated on an independent dataset of *P. aeruginosa* and the pipeline holds potential to be applied on other pathogenic bacteria as well.

## 2 Methods

### 2.1 Prediction schema

In this study, we tried to address two problems simultaneously; hence, our model works in two tiers. Firstly (referred to as Tier-I), it detects if the given genome belongs to antibiotic-resistant or susceptible *P. aeruginosa*. If the genome is predicted to belong to an antibiotic-resistant *P. aeruginosa*, the Tier-II prediction comes in, which predicts the antibiotic against which the *P. aeruginosa* was resistant.

### 2.2 Data sources and dataset preparation

**Figure 1.**
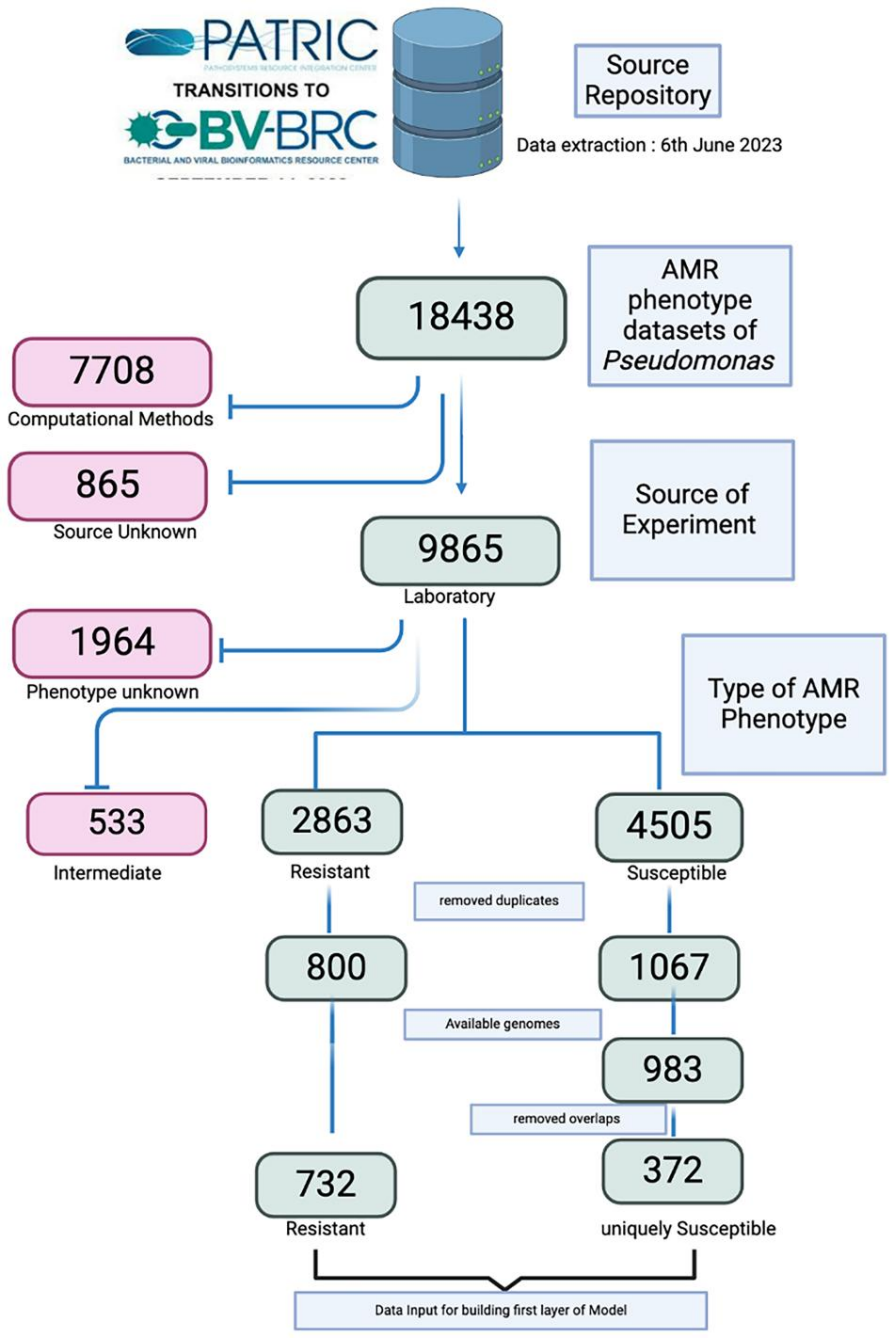
Preparation of input dataset for first layer of the machine learning model.

AMR phenotype data and corresponding genome sequences for *P. aeruginosa* isolates were retrieved on 6 June 2023, from the Pathosystems Resource Integration Center (PATRIC), part of the Bacterial and Viral Bioinformatics Resource Center (Olson *et al.* 2023). PATRIC provides detailed annotations for each isolate, including resistance phenotype (Resistant/Susceptible), type of evidence (Laboratory or Computational), and the specific antibiotics linked to resistance. For this study, we filtered the dataset to include only those isolates whose AMR information was supported by laboratory-based evidence (Fig. 1). Among the 18 438 *P. aeruginosa* isolates listed in the AMR phenotype section of PATRIC, the AMR phenotype of 7708 *P. aeruginosa* isolates was inferred using computational methods, while for 865 isolates, the source of AMR phenotype annotation was unknown. Laboratory-confirmed phenotype data were available for only 9865 isolates. Of these, 2863 isolates were categorized as resistant, 4505 as susceptible and 533 as intermediate. The AMR phenotype for the remaining 1964 isolates was not reported or remained unknown. Since the aim of Tier-I was to determine whether a *P. aeruginosa* isolate was antibiotic-resistant or susceptible, any organism showing resistance to even a single antibiotic was classified as resistant. Based on this criterion, we identified 732 *P. aeruginosa* isolates that were reported by PATRIC, as resistant to at least one antibiotic. Furthermore, an isolate was classified as sensitive only if it showed susceptibility to all the antibiotics considered in this study. Based on this criterion, we identified 372 unique isolates that met the condition and were therefore labeled as sensitive.

The aim of Tier-II was to predict the resistance or susceptibility of *P. aeruginosa* to specific antibiotics. Consequently, the approach to data preparation was modified accordingly. The selection of antibiotics commonly used to treat *P. aeruginosa* infections was adopted from the study of Ibrahim *et al.* (2020). From this list, we included only those antibiotics for which the PATRIC database contained resistance data from at least 100 *P. aeruginosa* isolates (Table 1, available as supplementary data at *Bioinformatics Advances* online).

### 2.3 Feature representation

**Generation of k-mers:** The genomic data of *P. aeruginosa* was encoded into k-mers using the Phenotypeseeker tool (Aun *et al.* 2018). To generate the k-mers, we utilized the GenomeTester4 software suite (Kaplinski *et al.* 2015), which offers a range of tools for extracting k-mers of specified lengths from genomic datasets. Specifically, we employed the GListMaker utility from GenomeTester4 to create k-mer profiles of all the genome samples. We generated binary k-mer lists for lengths 3, 8, 13, 18, 23, and 28 using a sliding window approach. However, codon boundaries were not incorporated while generating k-mers.

**Matrix construction:** After generating the k-mers, we created a binary matrix showing the presence or absence of each k-mer in each genome of *P. aeruginosa*. The presence/absence matrix was then used for statistical testing to find the association of k-mers with the phenotype.

Let *M* represent a matrix and *M_ij_* denote an entry in the row *i* and column *j* of that matrix. If a specific k-mer *k_j_* is present in a genome sample *i*, then the value of *M_ij_* will be 1; if it is absent, the value will be 0.

The matrix construction can be represented by the following equation:


Mij={0 if otherwise}

where *M* is the presence/absence matrix, *i* indicates the genome samples, and *j* indicates k-mers.

**Feature selection:** Selecting the most informative features is a critical step in developing an effective machine learning model. To identify relevant features, k-mers in the present case, we assessed the significance of different k-mers with the AMR phenotype. As part of this process, we first estimated the genomic distances between genomes of different antibiotic resistance phenotypes using Mash (Ondov *et al.* 2016). These distance estimates were then used to compute phylogeny-based weights with the help of the PyCogent python library (Knight *et al.* 2007).

**Testing the association of k-mers with the drug-resistance phenotype:** To determine the association of k-mers with drug-resistance phenotypes, a χ^2^ test was performed using the scipy.stats python package (Virtanen *et al.* 2020). During the chi-square test the null hypothesis assumed that there was no association between the k-mers and phenotype. In contrast, the alternative hypothesis assumed that the drug-sensitive and drug-resistant phenotype was associated with a specific pattern of k-mers.

Let χ^2^ denotes the chi-squared statistic. The null hypothesis *H*_0_ assumes no association between the k-mers and the binary phenotype whereas the alternative hypothesis *H*_1_ assumes that there is an association between the two. The test can be represented by the following equation:


$$\chi^{2}=\;\Sigma \frac{{\left(O_{i}-E_{i}\right)}^{2}}{O_{i}}$$

where *O_i_* represents the observed frequency of k-mer *i* in a specific phenotype, *E_i_* represents the expected frequency for that specific phenotype under the null hypothesis.

### 2.4 Machine learning models

To build the prediction model, we used three different machine learning algorithms, namely, support vector machine (SVM), decision trees (DT), and logistic regression (LR) with different k-mer lengths and evaluated their performance. All machine-learning algorithms were implemented using the python library, Scikit-learn (Pedregosa *et al.* 2011). After the initial screening of features explained in the Feature selection and optimization, we used only statistically significant k-mers for building the model, that is, only those k-mers whose *P* value was less than .05.

To select the number of statistically significant k-mers for our classification model, we performed lasso regression analysis to get 0.01%, 0.1%, 1%, and 5%. We performed this analysis with a maximum of 0.01%, 0.1%, 1%, and 5% lowest *P* value k-mers in different sets. During training, the datasets were split into training (75%) and test (25%) for each set. The proportion of class labels in the training and the test sets was kept the same as it was in the original, undivided dataset. The plots were made using the ggplot2 R library (Wickham 2016).

### 2.5 Cross-validation and performance evaluation

We evaluated the performance of various machine learning classifiers using metrics such as sensitivity, specificity, Matthews Correlation Coefficient (MCC) and the area under the receiver operating characteristic curve (AUC-ROC). Sensitivity, the true positive rate of prediction, was defined as:


$$\mathrm{Sensitivity}=(\frac{\mathrm{True}\;\mathrm{Positives}}{\mathrm{True}\;\mathrm{Positives}+\;\mathrm{False}\;\mathrm{Negatives}})*100$$

Specificity, that measures the true negative rate of prediction, was defined as:


$$\mathrm{Specificity}=(\frac{\mathrm{True}\;\mathrm{Negatives}}{\mathrm{True}\;\mathrm{Negatives}\;+\;\mathrm{False}\;\mathrm{Positives}})*100$$

The AUC-ROC represents the area under the receiver operating characteristic curve and indicates the model’s ability to distinguish between different classes. Its values range from 0 to 1, where 1 shows the perfect classification and 0 indicates no discriminatory power. These evaluation metrics provide an objective and unbiased means to assess the performance of a given classifier (Eng 2005, Fawcett 2006).

**SHAP analysis:** To understand the contribution of individual k-mers to the performance of the best-performing model, we used SHapley Additive exPlanations (SHAP) analysis. SHAP quantifies the impact of each feature on the model’s predictions (Chen *et al.* 2022). The SHAP explainer was constructed using the same datasets and parameters that were applied during model training and development. The resulting SHAP values for different k-mer lengths were analyzed to evaluate the influence of k-mer size on model performance and to identify the k-mers that contributed most significantly to the predictive power of the model.

**Independent dataset:** We validated our prediction model using an independent dataset from the AMRFinderPlus (version 3.11.11, database version 2023-04-17.1) of National Center for Biotechnology Information (NCBI) (Feldgarden *et al.* 2021). From AMRFinderPlus we selected only those genomes that were absent from the PATRIC repository at the time of retrieval (6 June 2023), ensuring their independence from the training data. The independent dataset consisted of 56 *P. aeruginosa* genomes, comprising 47 resistant and 9 susceptible isolates.

## 3 Results

### 3.1 Tier-I prediction

For model building, we first applied χ^2^ function to filter k-mers, retaining those with a *P* value <.05. From these, the top 1000 lowest *P*-valued k-mers were selected and used as features for different machine learning classifiers, namely, SVM, LR, and DT, to develop Tier-I prediction models. For LR model k-mer length 13, the maximum mean accuracy, sensitivity, specificity, and AUC-ROC values were 79%, 86%, 64%, and 0.75, respectively (Table 1). The obtained mean accuracy, sensitivity, specificity, and AUC-ROC values for the DT model k-mer length 13 were 79%, 84%, 70%, and 0.77, respectively (Table 1). The maximum mean accuracy, sensitivity, specificity, and AUC-ROC value of the SVM model of k-mer length 13 were 71%, 93%, 29%, and 0.61, respectively (Table 2, available as supplementary data at *Bioinformatics Advances* online). Our DT model with k-mer length 13 showed the most optimal performance. All reported performance metrics correspond to the independent 25% test set obtained from the random training-test split.

**Table 1.** Performance of LR and DT models created using different k-mer lengths for Tier-I (refer Table 2, available as supplementary data at *Bioinformatics Advances* online, for a comparative assessment).

K-mer size	Accuracy		Sensitivity		Specificity		AUC-ROC value		MCC value	
	LR	DT	LR	DT	LR	DT	LR	DT	LR	DT
8	68%	68%	98%	98%	11%	11%	0.54	0.54	0.18	0.18
13	79%	79%	86%	84%	64%	70%	0.75	0.77	0.52	0.53
18	72%	75%	85%	77%	48%	71%	0.66	0.74	0.35	0.47
23	71%	75%	85%	96%	45%	32%	0.65	0.64	0.32	0.39
28	73%	85%	85%	87%	50%	80%	0.67	0.83	0.37	0.66

**Figure 2.**
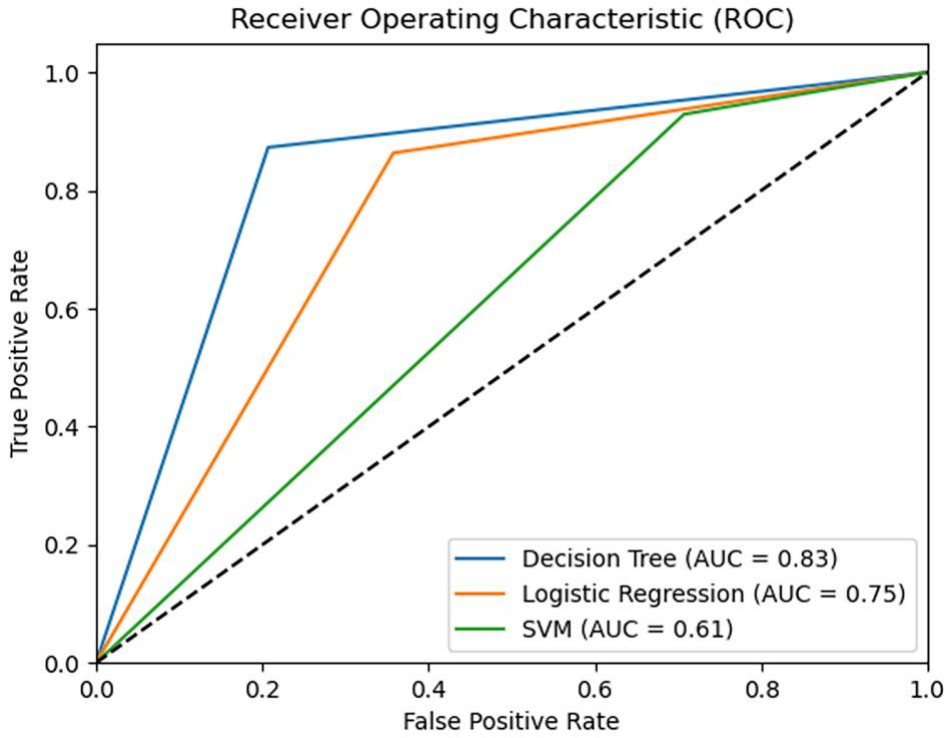
Plot showing ROC curves of three different machine learning classifiers used to develop Tier-I of prediction schema.

ROC Curve also showed that the performance of the DT model was better than other models (Fig. 2). We selected the DT model of k-mer length 13 for further analysis.

### 3.2 Tier-II predictions

In Tier-II, we developed antibiotic-specific decision tree models using k-mer length 13 (Table 2). Twenty-four antibiotics are commonly used to treat *P. aeruginosa* infections (Ibrahim *et al.* 2020). However, of these 24 antibiotics, we developed the prediction model only for 13 antibiotics. The reason was the availability of a very small number of genome sequences of *P. aeruginosa* in the PATRIC database, which were resistant to the remaining antibiotics. The antibiotics’ class for which we developed prediction models were Penicillins and Penicillin + β-lactamase inhibitor combination (Piperacillin), Cephalosporins (Ceftazidime, Cefepime), Monobactams (Aztreonam), Fluoroquinolones (Ciprofloxacin, Levofloxacin), Carbapenems (Doripenem, Imipenem, and Meropenem), Novel β-lactams and β-lactamase inhibitors (Ceftolozane) and Aminoglycosides (Amikacin, Gentamycin, and Tobramycin). During the training of DT models for the prediction of genomes that were resistant for these 13 antibiotics we kept the k-mer size as 13 and chose the top 1000 k-mers, same as in the Tier-I. As shown in Table 2, the maximum and minimum prediction accuracy was obtained with Imipenem and Aztreonam, which were 0.98 and 0.71, respectively. Antibiotics class-wise analysis revealed that the maximum accuracy was achieved in Piperacillin 0.98 followed by Carbapenems. Among the three antibiotics that were part of class Carbapenems, maximum accuracy was obtained in Imipenem (0.98) followed by Doripenem (0.95) and Meropenem (0.86). In the three classes of Aminoglycosides namely Amikacin, Gentamycin, and Tobramycin, we obtained a similar accuracy of 0.91-0.93. In antibiotic class Fluoroquinolones, Ciprofloxacin showed comparatively better accuracy of 0.92 than the Levofloxacin where accuracy was 0.85. Additionally, we evaluated how the number of top ranked k-mers, selected on the basis of lowest *P* value, affected the model’s performance. For this we used DT classifier at k-mer length 13 and developed models using top 0.01%, 0.1%, 1%, 5%, and 10% lowest *P* value k-mers both for Tier-I (Table 3) and Tier-II (Table 2, available as supplementary data at *Bioinformatics Advances* online). All DT models developed earlier, were using the top 1000 k-mers which is the default mode of learning in Phenotypeseeker (Aun *et al.* 2018). We found that top 1% k-mers of DT models developed using k-mer length 13 yielded the most optimal performance for Tier-I (Table 3). We observed a gradual increase in the model’s performance as the top k-mers were increased from 1% to 5% for most antibiotics at our Tier-II (Table 2, available as supplementary data at *Bioinformatics Advances* online). This variation in behaviour of model performance at Tier-I and Tier-II indicates that with the increase in specificity of dataset more significant k-mers are available for model construction.

**Table 2.** Performance of DT model used on antibiotics in the Tier-II of our resistance prediction model with k-mer length 13.

Antibiotic class	Antibiotic	No. of genomes	Accuracy	Sensitivity	Specificity	MCC value	AUC-ROC value
Penicillins and penicillin + β-lactamase inhibitor combination	Piperacillin	400	93%	78%	100%	0.84	0.89
Cephalosporins	Ceftazidime	1096	82%	67%	94%	0.64	0.80
	Cefepime	268	75%	73%	77%	0.50	0.75
Monobactams	Aztreonam	275	71%	64%	77%	0.42	0.71
Fluoroquinolones	Ciprofloxacin	781	92%	86%	98%	0.85	0.92
	Levofloxacin	761	85%	76%	93%	0.71	0.85
Carbapenems	Doripenem	91	95%	92%	10%	0.88	0.96
	Imipenem	406	98%	97%	99%	0.96	0.98
	Meropenem	1061	86%	81%	93%	0.73	0.87
Novel β-lactams and β-lactamase inhibitors	Ceftolozane	40	No k-mers generated				
Aminoglycosides	Amikacin	769	91%	57%	99%	0.67	0.78
	Gentamycin	377	91%	81%	95%	0.79	0.88
	Tobramycin	753	93%	82%	98%	0.83	0.90

**Table 3.** Performance of DT models of k-mer length 13 developed by selecting different top k-mers (lowest *P* value) for Tier-I.

No. unique k-mers	Accuracy	Sensitivity	Specificity	AUC-ROC value	MCC value
1000 (∼0.001%)	79%	84%	70%	0.77	0.53
6710 (0.01%)	76%	95%	40%	0.67	0.43
67 108 (0.1%)	77%	97%	36%	0.67	0.46
671 088 (1%)	85%	77%	99%	0.88	0.72
3 355 443 (5%)	83%	76%	95%	0.86	0.67
6 710 886 (10%)	83%	77%	95%	0.86	0.68

### 3.3 Model interpretation

**Figure 3.**
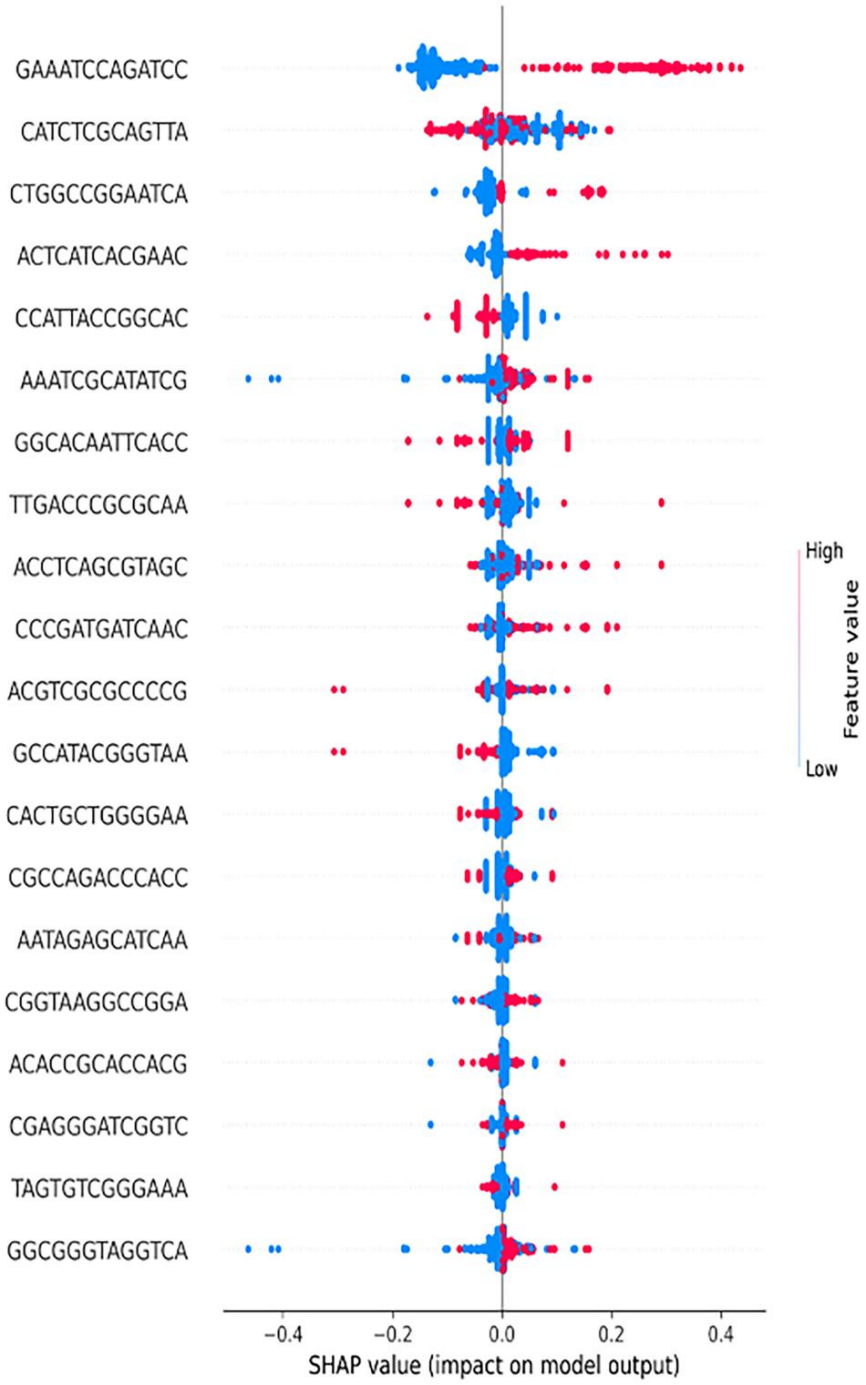
SHAP Summary Plot evaluated by the SHAP method and the effects of each feature on the outcome of DT model of k-mer length 13 (Tier-I) Pink color represents high feature value, i.e. strong presence of that particular k-mer in resistant strain if the value is positive and in sensitive strains if the value is negative. Similarly, blue color represents low feature value, i.e. absence of that particular k-mer in resistant strain if the value is positive and in sensitive strain if the value is negative.

**Figure 4.**
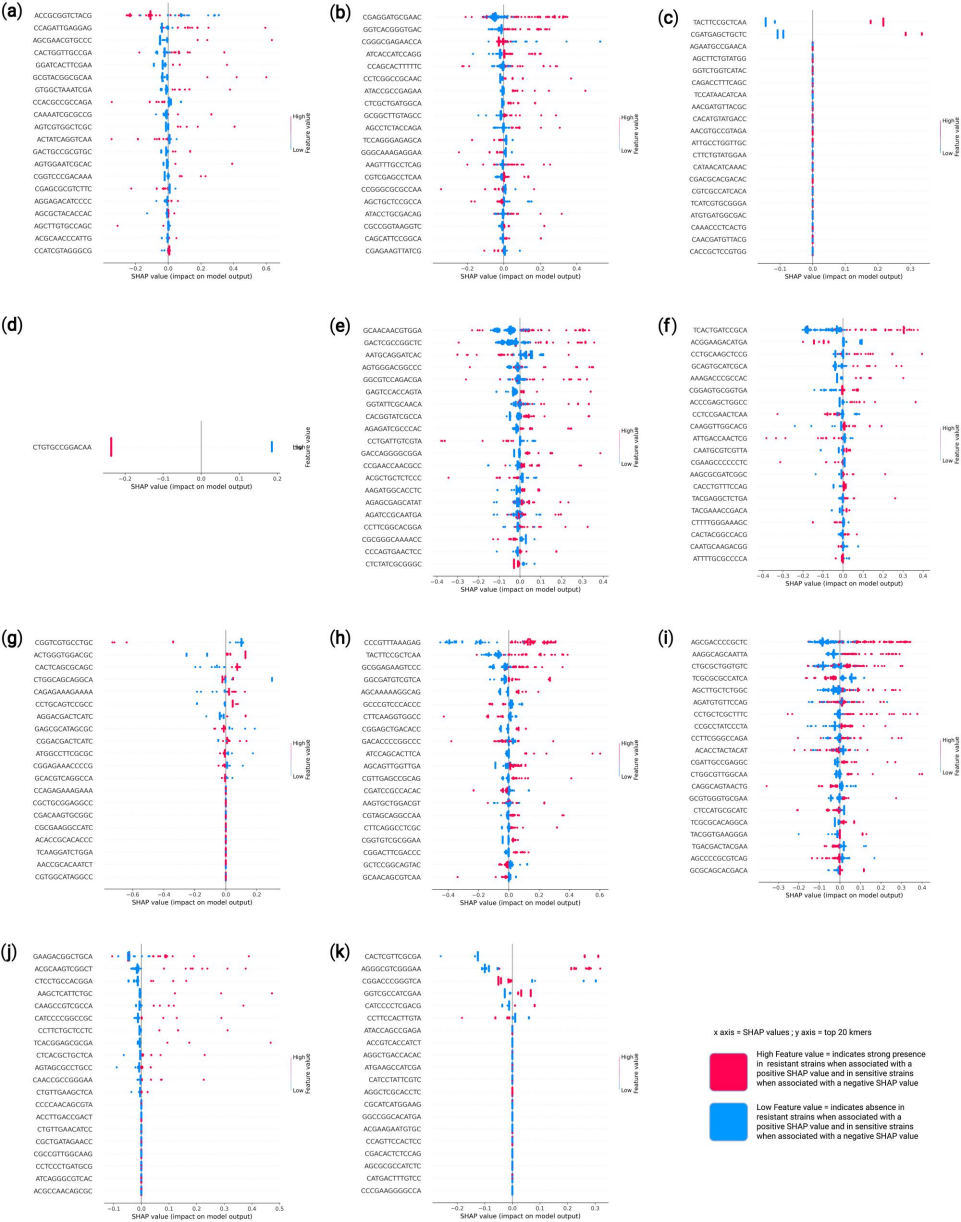
SHAP analysis of Tier-II decision tree models trained on 13-mer features for each antibiotic. Pink color represents high feature value i.e. strong presence of that particular k-mer in resistant strain if the value is positive and in sensitive strains if the value is negative. Similarly, blue color represents low feature value i.e. absence of that particular k-mer in resistant strain if the value is positive and in sensitive strain if the value is negative. (a) Piperacillin, (b) Ceftazidime, (c) Cefepime, (d) Aztreonam, (e) Ciprofloxacin, (f) Levofloxacin, (g) Doripenem, (h) Imipenem, (i) Meropenem, (j) Amikacina, and (k) Gentamycin. (Full-size plots for all antibiotics are provided in Figures 1–11, available as supplementary data at *Bioinformatics Advances* online.)

The SHAP summary plot discerns the contribution of individual k-mers to the predictions of the trained model. Each point represents a SHAP value for a specific k-mer in a single sample, with pink indicating a high feature value (indicating frequent presence of the k-mer) and blue indicating a low feature value (rare presence or absence). We have constructed such SHAP plots to interpret the findings of our model at both Tier-I (Fig. 3) and Tier-II (Fig. 4). In these plots, the horizontal position of the points reflects the SHAP value—how much that k-mer pushed the model output towards a positive or negative class. For instance, in our SHAP interpretation plot for Tier-I (Fig. 3) k-mers ACGTCGCGCCCCG and GCCATACGGGTAA exhibited a strong negative impact on model output when present in high abundance (pink dots on the left), which suggest their association with the negative class. Conversely, k-mers such as GAAATCCAGATCC and ACTCATCACGAAC possessed SHAP values that shifted towards the positive side when their abundance was higher, indicating a contribution towards the positive class. Some k-mers like TAGTGTCGGGAAA exhibited minimal spread around zero, implying limited contribution to the model’s predictions. A few k-mers, such as CATCTCGCAGTTA, exhibited a wider SHAP value spread, indicating variability in their contribution across samples. The plot reveals that not all high-frequency k-mers were equally informative; their impact depended on both frequency and context within the model. It is also evident that certain k-mers consistently aligned with either positive or negative predictions, supporting their potential biological relevance. Overall, this SHAP plot is a powerful tool for interpreting the k-mer patterns that most strongly influenced the model’s classification decisions. We then interpreted our Tier-II, antibiotic specific DT models of k-mer length 13 (Fig. 4).

#### 3.3.1 Class—penicillins

The SHAP analysis of the DT model developed to predict Piperacillin (Fig. 4a) resistance in *P. aeruginosa* depicted k-mer ACCGCGGTCTACG with a lot of pink dots on the left side of SHAP plot. This indicated that the k-mer ACCGCGGTCTACG had a strong negative impact on model output when present in high abundance, suggesting its association with the penicillin-sensitive *P. aeruginosa*. On the contrary, k-mers such as CCAGATTGAGGAG and CACTGGTTGCCGA exhibited SHAP values that shifted towards the positive side when their abundance was higher, indicating a contribution towards the positive class.

#### 3.3.2 Class—cephalosporins

In case of Ceftazidime (Fig. 4b), CGAGGATGCGAAC and GGTCACGGGTGAC showed strong negative SHAP values when its feature value was low (blue) and strong positive SHAP values when its feature value was high (pink) indicating that its presence pushed the prediction towards resistance and absence pushed it towards sensitivity. Thereby, making it a strong marker for resistance. On the other hand, for Cefepime (Fig. 4c), only two k-mers (TACTTCCGCTCAA and CGATGAGCTGCTC) showed effect on prediction. Remaining 18 k-mers were neutral in nature and did not show any significant impact on either of the predictions. TACTTCCGCTCAA and CGATGAGCTGCTC showed lower impact on negative prediction when absent and higher impact on positive prediction when present. Therefore, they serve as markers for resistance.

#### 3.3.3 Class—monobactams

The SHAP plot for Aztreonam (Fig. 4d), depicts only a single k-mer (CTGTGCCGGACAA), playing a significant role in both positive and negative predictions. The SHAP plot also indicated that the presence of this k-mers was related to the drug-sensitivity and its absence with drug resistance. Since, the prediction was dependent on the presence of one k-mer only, it might be the underlying reason for the low predictive performance of Aztreonam resistant *P. aeruginosa*.

#### 3.3.4 Class—fluoroquinolones

For Ciprofloxacin (Fig. 4e), absence of k-mers GCAACAACGTGGA and GACTCGCCGGCTC could be considered as strong markers of negative prediction. Interestingly, high presence of k-mer CTCTATCGCGGGC is indicative of sensitive *P. aeruginosa* or negative prediction and absence indicates resistant *P. aeruginosa* or positive prediction. On the other hand, for Levofloxacin (Fig. 4f) absence of k-mer TCACTGATCCGCA has a high negative impact on the model’s prediction and presence holds a significant positive impact indicating it to be a strong marker of Levofloxacin resistance in *P. aeruginosa*.

#### 3.3.5 Class—carbapenems

In the case of Doripenem (Fig. 4g), absence of k-mer CGGTCGTGCCTGC had a positive impact on predictions of resistant *P*. *aeruginosa*. This indicated its absence in the drug-resistant *P. aeruginosa*. On the other hand, for Imipenem (Fig. 4h), k-mer CCCGTTTAAAGAG had a strong impact on positive predictions and its absence had a strong impact on negative predictions indicating that it could act as a reliable resistance marker. Similarly, for Meropenem (Fig. 4i), k-mer AGCGACCCCGCTC held a high positive impact on model predictions and its absence had a strong negative impact indicating its reliability as a resistance marker.

#### 3.3.6 Class—aminoglycosides

Interestingly for Amikacin (Fig. 4j), absence of the majority of top 20 k-mers has a negative impact on model predictions. It indicates lack of distinct resistance markers and scattered presence of significant resistance markers in *P. aeruginosa*. On the other hand for Gentamycin (Fig. 4k), presence of k-mer CGGACCCGGGTCA had a negative impact on model predictions indicating its presence in sensitive *P. aeruginosa*. Whereas, presence of GGTCGCCATCGAA had a positive impact on model predictions indicating its presence in resistant *P. aeruginosa*. The SHAP plot for Tobramycin could not be obtained.

### 3.4 Validation of the model

We validated the Tier-I of our model using an independent dataset. Of the 47 resistant genomes tested, all were correctly identified as resistant and of the 9 susceptible genomes tested, 8 were identified as susceptible. Overall, we got an accuracy of 98% in our validation. We could not validate the Tier-II models because the genomes retrieved from NCBI-AMR Finder were not annotated with the information about the specific antibiotics.

### 3.5 Genomic mapping of significant k-mers

We further mapped the top-20 k-mers that were labelled as the most significant by SHAP analysis in determining the output of the trained models to the genomes that we used to train the model (Table 4, available as supplementary data at *Bioinformatics Advances* online). In Tier-I out of 20 k-mers, only 12 k-mers were aligned to the genic region. In Tier-II the number of aligned k-mers range from 7 to 15 for various antibiotic specific models. The aligned k-mers were mapped to genes that belong to virulence factors, heavy metal processing, and other vital processes.

## 4 Discussion

Reports of chronic infections in humans, classification as a priority one pathogen by WHO and development of multidrug-resistant strains has multiplied the attention on *P. aeruginosa*. The identification of resistance phenotypes is an essential prerequisite for all clinical procedures but traditional methods for detecting AMR are often time-consuming and limited in scope. The development of data backed machine learning based drug-phenotype prediction methods are not only essential but also indispensable in this scenario given the growing global threat posed by antibiotic resistant strains, particularly *P. aeruginosa*. In this work, we have reported the development of an interpretable machine learning tool using k-mers of 13 nucleotides. The proposed ML framework works in two tiers. Tier-I distinguishes resistant versus susceptible isolates of *P. aeruginosa*, while Tier-II identifies 13 specific antibiotic(s) associated with resistance. We used three different ML classifiers, namely LR, SVM, and DT. The performance of model was evaluated using parameters like sensitivity, specificity, accuracy, MCC and AUC-ROC Value. Among all three ML methods, the maximum performance was achieved by DT Classifier with a mean accuracy of 79%, sensitivity of 84%, specificity of 70%, and AUC-ROC of 0.77. Validation on an independent dataset was performed for Tier-I with an accuracy of 98% indicates the efficiency and robustness of the model. Targeted resistance prediction and potential utility of the model in clinical or surveillance settings can be improved with further validation of Tier-II models as more data becomes available. In order to understand the relative importance of different genomic k-mers in prediction, we also incorporated SHAP analysis.

This analysis provided the top 20 discriminative k-mers from both Tier-I and Tier-II models, representing k-mers that have the highest impact on model predictions. Genomic mapping of these k-mers revealed associations not limited to annotated AMR determinants, suggesting involvement of genomic elements other than the known antibiotic resistance genes in mediating resistance. While the results demonstrate the utility of k-mer-based prediction of resistance, we acknowledge the inherent limitations of genome-only approaches. We propose that this framework provides a scalable and rapid strategy to augment resistance prediction in *P. aeruginosa* beyond canonical AMR gene profiling.

## Supplementary Material

vbaf190_Supplementary_Data

## Data Availability

The data underlying this article are available in PATRIC repository of BV-BRC at https://www.bv-brc.org/view/Taxonomy/286#view_tab=amr.
