# Supplementary material for: An explainable machine learning pipeline for prediction of antimicrobial resistance in Pseudomonas aeruginosa
Source: Bioinform Adv. 2025 Aug 22;5(1):vbaf190. doi: 10.1093/bioadv/vbaf190 (PMC12380447; doi:10.1093/bioadv/vbaf190)
Supplement: vbaf190_Supplementary_Data [file vbaf190_supplementary_data.docx]

Supplementary Table 1: Summary of antibiotic-specific AMR phenotype datasets available in the PATRIC database. The listed antibiotics are commonly used for treating P. aeruginosa infections as per Ibrahim et. al (Ibrahim et al., 2020)

| **Antibiotic class** | **Antibiotic** | **Number of genomes** |
| --- | --- | --- |
| Penicillins and penicillin+β-lactamase inhibitor combination | Piperacillin | 400 |
|  | Ticarcillin/ clavulanate | 3 |
| Cephalosporins | Ceftazidime | 1096 |
|  | Cefepime | 268 |
|  | Cefiderocol | 0 |
| Monobactams | Aztreonam | 275 |
| Fluoroquinolones | Ciprofloxacin | 781 |
|  | Levofloxacin | 761 |
|  | Delafloxacin | 0 |
|  | Finafloxacin | 0 |
| Phosphonic acid derivative | Fosfomycin | 27 |
| Carbapenems | Doripenem | 91 |
|  | Imipenem/cilastatin | 406 |
|  | Meropenem | 1061 |
| Novel β-lactams and β-lactamase inhibitors | Ceftazidime/avibactam | 95 |
|  | Ceftolozane/tazobactam | 40 |
|  | Imipenem/cilastatin– relebactam | 6 |
|  | Meropenem– vaborbactam | 6 |
| Aminoglycosides | Tobramycin | 753 |
|  | Gentamycin | 377 |
|  | Amikacin | 769 |
|  | Plazomicin | 0 |
| Polymyxins | Colistin | 618 |
|  | Polymyxin B | 85 |

Supplementary Table 2: Comparative Performance of SVM, LR and DT Models trained using different k-mer lengths (Tier 1)

| K-mer size | Accuracy | | | Sensitivity | | | Specificity | | | AUC-ROC value | | | MCC  Value | | |
| --- | --- | --- | --- | --- | --- | --- | --- | --- | --- | --- | --- | --- | --- | --- | --- |
|  | SVM | LR | DT | SVM | LR | DT | SVM | LR | DT | SVM | LR | DT | SVM | LR | DT |
| 8 | 68% | 68% | 68% | 98% | 98% | 98% | 11% | 11% | 11% | 0.54 | 0.54 | 0.54 | 0.18 | 0.18 | 0.18 |
| 13 | 71% | 79% | 79% | 93% | 86% | 84% | 29% | 64% | 70% | 0.61 | 0.75 | 0.77 | 0.30 | 0.52 | 0.53 |
| 18 | 72% | 72% | 75% | 88% | 85% | 77% | 40% | 48% | 71% | 0.64 | 0.66 | 0.74 | 0.33 | 0.35 | 0.47 |
| 23 | 72% | 71% | 75% | 90% | 85% | 96% | 34% | 45% | 32% | 0.62 | 0.65 | 0.64 | 0.31 | 0.32 | 0.39 |
| 28 | 72% | 73% | 85% | 89% | 85% | 87% | 38% | 50% | 80% | 0.64 | 0.67 | 0.83 | 0.32 | 0.37 | 0.66 |

Supplementary Table 3: Performance of Tier 2 Decision Tree models developed using different top-ranked 13-mer k-mers (p<0.05)

| Antibiotic Class | Antibiotic | No. of k-mers selected | Accuracy | Sensitivity | Specificity | MCC Value | AUC-ROC Value |
| --- | --- | --- | --- | --- | --- | --- | --- |
| Penicillins and penicillin+β-lactamase inhibitor combination | Piperacillin | 1000 | 93% | 78% | 1.0 | 0.84 | 0.89 |
|  |  | 0.01% | 98% | 93% | 1.0 | 0.95 | 0.97 |
|  |  | 0.1% | 87% | 66% | 0.97 | 0.69 | 0.81 |
|  |  | 1% | 90% | 77% | 0.96 | 0.77 | 0.87 |
|  |  | 5% | 91% | 81% | 0.95 | 0.78 | 0.88 |
|  |  | 10% | 85% | 54% | 0.98 | 0.63 | 0.76 |
| Cephalosporins | Ceftazidime | 1000 | 76% | 60% | 0.88 | 0.51 | 0.74 |
|  |  | 0.01% | 84% | 72% | 0.93 | 0.67 | 0.82 |
|  |  | 0.1% | 73% | 59% | 0.84 | 0.45 | 0.72 |
|  |  | 1% | 76% | 67% | 0.83 | 0.5 | 0.75 |
|  |  | 5% | 76% | 67% | 0.83 | 0.5 | 0.75 |
|  |  | 10% | 76% | 67% | 0.83 | 0.5 | 0.75 |
|  | Cefepime | 1000 | 75% | 73% | 0.77 | 0.5 | 0.75 |
|  |  | 0.01% | 75% | 73% | 0.77 | 0.5 | 0.75 |
|  |  | 0.1% No k-mers generated | | | | | |
| Monobactams | Aztreonam | 1000 | 71% | 64% | 0.77 | 0.42 | 0.71 |
|  |  | 0.01 | 71% | 64% | 0.77 | 0.42 | 0.71 |
|  |  | 0.1% No k-mers generated | | | | | |
| Fluoroquinolones | Ciprofloxacin | 1000 | 92% | 86% | 0.98 | 0.85 | 0.92 |
|  |  | 0.01% | 88% | 84% | 0.92 | 0.76 | 0.88 |
|  |  | 0.1% | 88% | 84% | 0.92 | 0.76 | 0.88 |
|  |  | 1% | 88% | 87% | 0.89 | 0.76 | 0.88 |
|  |  | 5% | 88% | 87% | 0.89 | 0.76 | 0.88 |
|  |  | 10% | 88% | 87% | 0.89 | 0.76 | 0.88 |
|  | Levofloxacin | 1000 | 85% | 76% | 0.93 | 0.71 | 0.85 |
|  |  | 0.01% | 90% | 83% | 0.96 | 0.8 | 0.89 |
|  |  | 0.1% | 90% | 83% | 0.96 | 0.8 | 0.89 |
|  |  | 1% | 92% | 87% | 0.97 | 0.84 | 0.92 |
|  |  | 5% | 92% | 87% | 0.97 | 0.84 | 0.92 |
|  |  | 10% | 95% | 91% | 0.98 | 0.89 | 0.94 |
| Carbapenems | Doripenem | 1000 | 95% | 92% | 0.1 | 0.88 | 0.96 |
|  |  | 0.01% | No k-mers generated | | | | |
|  | Imipenem | 1000 | 98% | 97% | 0.99 | 0.96 | 0.98 |
|  |  | 0.01% | 88% | 93% | 0.84 | 0.77 | 0.88 |
|  |  | 0.1% | 99% | 98% | 0.99 | 0.98 | 0.99 |
|  |  | 1% | 88% | 93% | 0.84 | 0.77 | 0.88 |
|  |  | 5% | 100% | 100% | 0.99 | 0.99 | 1.0 |
|  |  | 10% | 99% | 99% | 0.99 | 0.99 | 0.99 |
|  | Meropenem | 1000 | 86% | 81% | 0.93 | 0.73 | 0.87 |
|  |  | 0.01% | 73% | 72% | 0.74 | 0.46 | 0.73 |
|  |  | 0.1% | 72% | 72% | 0.72 | 0.44 | 0.72 |
|  |  | 1% | 72% | 72% | 0.72 | 0.44 | 0.72 |
|  |  | 5% | 72% | 72% | 0.72 | 0.44 | 0.72 |
|  |  | 10 | 72% | 72% | 0.72 | 0.44 | 0.72 |
| Novel β-lactams and β-lactamase inhibitors | Ceftolozane | NOT RUNNING | | | | | |
| Aminoglycosides | Amikacin | 1000 | 0.91 | 57% | 0.99 | 0.67 | 0.78 |
|  |  | 0.01% | 0.94 | 68% | 1 | 0.79 | 0.84 |
|  |  | 0.1% | 97% | 84% | 1 | 0.89 | 0.92 |
|  |  | 1% | 93% | 69% | 0.97 | 0.72 | 0.83 |
|  |  | 5% | 99% | 92% | 1 | 0.95 | 0.96 |
|  |  | 10% | 99% | 94% | 1 | 0.96 | 0.97 |
|  | Gentamycin | 1000 | 91% | 81% | 0.95 | 0.79 | 0.88 |
|  |  | 0.01% | 91% | 83% | 0.94 | 0.77 | 0.89 |
|  |  | 0.1% | 91% | 84% | 0.94 | 0.79 | 0.89 |
|  |  | 1% | 92% | 83% | 0.95 | 0.8 | 0.89 |
|  |  | 5% | 92% | 83% | 0.95 | 0.8 | 0.89 |
|  |  | 10% | 92% | 83% | 0.95 | 0.8 | 0.89 |
|  | Tobramycin | 1000 | 93% | 82% | 0.98 | 0.83 | 0.9 |
|  |  | 0.01% | 95% | 88% | 0.98 | 0.88 | 0.93 |
|  |  | 0.1% | 93% | 86% | 0.96 | 0.83 | 0.91 |
|  |  | 1% | 92% | 86% | 0.94 | 0.81 | 0.9 |
|  |  | 5% | 92% | 86% | 0.94 | 0.81 | 0.9 |
|  |  | 10% | 92% | 86% | 0.94 | 0.81 | 0.9 |

Supplementary Table 4: Top 20 kmers of Tier I and II mapped to *P .aeruginosa* genomes using the BLASTn program against the clustered NR database specifying the organism as *P. aeruginosa .* Genomic coordinates of the subject sequences have been specified. Mapped genes were highlighted in bold, along with their protein names.

| Kmers | Tier-1 | Tier-2 | | | | | | | | | |
| --- | --- | --- | --- | --- | --- | --- | --- | --- | --- | --- | --- |
|  |  | Amikacin | Cefepime | Ceftazidime | Ciprofloxacin | Doripenem | Gentamycin | Imipenem | Levofloxacin | Meropenem | piperacillin |
| 1 | **intI1**- class 1 integron integrase, **xerC_2**- tyrosine recombinase | **tnp**- site-specific recombinase/resolvase family | **intI1**- class 1 integron integrase,**xerD**- tyrosine recombinase | **intI1**- class 1 integron integrase,**xerD-** tyrosine recombinase | **yebA3**- Putative peptidase | NA | **intI1**- class 1 integron integrase | NA | **intI**- integrase, **xerD**- tyrosine recombinase | **folP_1**- dihydropteroate synthase,**sulI-** dihydropteroate synthase | **gyrA-** DNA gyrase subunit A |
| 2 | NA | NA | NA | **bcsQ**- Cellulose biosynthesis protein, **soj_5-** Sporulation initiation inhibitor protein soj, **parA-** partitioning protein ParA | NA | NA | **intI**- integrase, **xerD**- tyrosine recombinase | **intI**- integrase, **xerD-** tyrosine recombinase | **PA14**- polysaccharide biosynthesis protein, **wbpM**- nucleotide sugar epimerase/dehydratase WbpM, **capD-** nucleotide sugar epimerase/dehydratase WbpM | **qacEdelta1**- quaternary ammonium compound resistance protein, **qacED1-** quaternary ammonium compound efflux SMR transporter | **tniA-** transposase |
| 3 | **potF_1**- periplasmic polyamine binding protein, **spuD_1-** Putrescine-binding periplasmic protein | NA | **folP_2**- dihydropteroate synthase, **sulI**- dihydropteroate synthase | NA | NA | **folP_1**- dihydropteroate synthase, **sulI-** dihydropteroate synthase | NA | NA | NA | **prs-**phosphoribosylpyrophosphate synthetase | NA |
| 4 | **wapB**-1,2-glucosyltransferase | NA | **intI1**- class 1 integron integrase,**xerD**- tyrosine recombinase | **fimU**- type 4 fimbrial biogenesis protein FimU | NA | NA | NA | NA | NA | **pdp**- thymidine phosphorylase | NA |
| 5 | **nasA**-Nitrate transporter, **narK**- nitrite transporter family protein | **fepA**- Outer membrane receptor for ferrienterochelin and colicins | **intI1**- class 1 integron integrase | NA | NA | **qacEdelta1**- quaternary ammonium compound efflux SMR transporter | NA | **qacEdelta1**- quaternary ammonium compound resistance protein | NA | **cdiA**- cdiA, **hpmA1-** hemagg_act and Fil_hemagg_2 domain-containing protein | **ygiN-** Quinol monooxygenase YgiN, **lsrG_2-** antibiotic biosynthesis monooxygenase |
| 6 | NA | NA | **intI1**- class 1 integron integrase | **ftsH**- ATP-binding protein | NA | **folP_1**- dihydropteroate synthase, **sulI**- dihydropteroate synthase | NA | **tniB**- TniB NTP-binding protein | NA | NA | NA |
| 7 | NA | NA | NA | NA | NA | NA | NA | **virB4**- conjugative transfer ATPase | **tsaD**- tRNA (adenosine(37)-N6)-threonylcarbamoyltransferase complex transferase subunit TsaD,**gcp-** O-sialoglycoprotein endopeptidase | NA | **aacA4**- aminoglycoside N(6')-acetyltransferase type 1, **aac(6')-Ib-** AAC(6')-Ib family aminoglycoside 6'-N-acetyltransferase,**aacA4'-17-** Aminoglycoside 6'-N-acetyltransferase |
| 8 | **rve**- Viral genome integrase, **tniA**- TniA protein, **tnpA**- TnpA | NA | NA | **pilB**- type IV-A pilus assembly ATPase PilB,**xpsE**- Type II secretion system protein E | **spuD_3**- Putrescine-binding periplasmic protein | **folP_1**- dihydropteroate synthase, **sulI**- dihydropteroate synthase | NA | NA | NA | NA | **tnpA**- IS6100 transposase |
| 9 | **OruR**- ornithine utilization transcriptional regulator , **virS_5**- HTH-type transcriptional regulator VirS | **mobH**- MobH family relaxase | **intI1**- class 1 integron integrase, **xerD**- tyrosine recombinase | NA | **tniR**- transposase | NA | NA | NA | NA | NA | **aadA2-** aminoglycoside-3''-adenylyltransferase |
| 10 | **adhB_1**- Alcohol dehydrogenase cytochrome c subunit | NA | **intI1**- class 1 integron integrase | **ssb**- single-stranded DNA-binding protein | **mlaC**- putative phospholipid-binding protein MlaC | NA | NA | NA | NA | NA | NA |
| 11 | **smc_3**- Chromosome partition protein Smc | **tniB**- putative ATP-binding protein | **qacEdelta1**-quaternary ammonium compound resistance protein | NA | **mdpA**- metallopeptidase MdpA | **rmd**- GDP-6-deoxy-D-mannose reductase | **yejE_2**- Inner membrane ABC transporter permease protein yejE | NA | **wbpM**- nucleotide sugar epimerase/dehydratase WbpM, **pglF**- UDP-N-acetyl-alpha-D-glucosamine C6 dehydratase | NA | NA |
| 12 | NA | **tnpA**- transposase | **intI1**- class 1 integron integrase, **xerD**- tyrosine recombinase | NA | **cdiA_1**- tRNA nuclease CdiA, **hpmA1-** hemagg_act and DUF637 domain-containing protein | NA | NA | NA | **waaL**- O-antigen ligase WaaL | NA | NA |
| 13 | **topB_1**- DNA topoisomerase III | NA | NA | NA | **rdmC_2**- Aclacinomycin methylesterase RdmC | **qacEdelta1**- quaternary ammonium compound efflux SMR transporter | **tnpA**- TnpA | NA | **btuB_1**- TonB-dependent outer membrane receptor | NA | **csrA**- carbon storage regulator CsrA |
| 14 | **ccmE**- cytochrome c maturation protein CcmE | NA | **intI1**- class 1 integron integrase, **xerD**- tyrosine recombinase | NA | **dnaK_1**- Chaperone protein DnaK | **qacEdelta1**- quaternary ammonium compound resistance protein | NA | **aspS**- aspartate--tRNA ligase | **aspC-** Aspartate aminotransferase | NA | **topA**- type 1DNA topoisomerase, **topA2**- DNA topoisomerase |
| 15 | NA | **tauB**- Taurine import ATP-binding protein TauB | **intI1-** class 1 integron integrase, **xerD**- tyrosine recombinase | NA | NA | **glmM**- phosphoglucosamine mutase | **cmlA9**- Chloramphenicol efflux protein, **floR**- chloramphenicol/florfenicol efflux MFS transporter FloR2 | **parC**- DNA topoisomerase IV subunit A | NA | NA | **folP_1**- dihydropteroate synthase, **sulI-** dihydropteroate synthase |
| 16 | NA | **merR**- mercury resistance regulatory protein | **intI1**- class 1 integron integrase , **xerD**- tyrosine recombinase | **grsB**- Pyoverdine sidechain non-ribosomal peptide synthetase, **pvdJ**- Pyoverdine sidechain non-ribosomal peptide synthetase PvdJ | NA | NA | **prmB_1**-50S ribosomal protein L3 glutaminemethyltransferase | **htpG**- molecular chaperone HtpG | **aprF**- alkaline protease secretion protein AprF, **tolC_2-** Outer membrane protein TolC | **copD**- copper homeostasis membrane protein CopD, **yebZ**- copper resistance protein D | NA |
| 17 | NA | NA | **intI1**- class 1 integron integrase, **xerD**- tyrosine recombinase | **hsdR**- Type I restriction enzyme R protein | **merA**- mercury(II) reductase | **intI1**- class 1 integron integrase IntI1 | NA | **folP_1**- dihydropteroate synthase, **sulI**- dihydropteroate synthase | NA | NA | **nqrC**- Na+-translocating NADH:ubiquinone oxidoreductase subunit Nrq3 |
| 18 | **topB**- DNA topoisomerase 3 | NA | **intI1**- class 1 integron integrase, **xerD-** tyrosine recombinase | **hsdR**- Type I restriction enzyme R protein | NA | **intI1**- class 1 integron integrase, **xerD**- tyrosine recombinase | **recD_1**- RecBCD enzyme subunit RecD | NA | NA | NA | **tet(G)-** tetracycline efflux MFS transporter Tet(G) |
| 19 | NA | **cysE**- serine O-acetyltransferase | NA | NA | NA | **folP_1**- dihydropteroate synthase, **sulI-** dihydropteroate synthase | NA | NA | NA | **nucC**- CBASS effector endonuclease NucC | NA |
| 20 | **gldG**- BC-type putative transport system involved in gliding motility, auxiliary component | **pbuE_4**- Purine efflux pump PbuE | **intI1**- class 1 integron integrase, **xerD**- tyrosine recombinase | NA | **prkC_2**- Serine/threonine-protein kinase PrkC | **tnpA**- IS6100 transposase | NA | **tniB delta**- transposase | NA | NA | **bkdC**- Dihydrolipoyllysine-residue acyltransferase component of branched-chain alpha-ketoacid dehydrogenase complex |

**Supplementary Figures**

**SHAP Plots of Antibiotic specific models of Decision Tree Classifier and k-mer length 13 (Tier - 2) (Full size figures of Fig 4 main manuscript)**

Penicillins and penicillin+β-lactamase inhibitor combination

**Supplementary Fig 1: PIPERACILLIN**


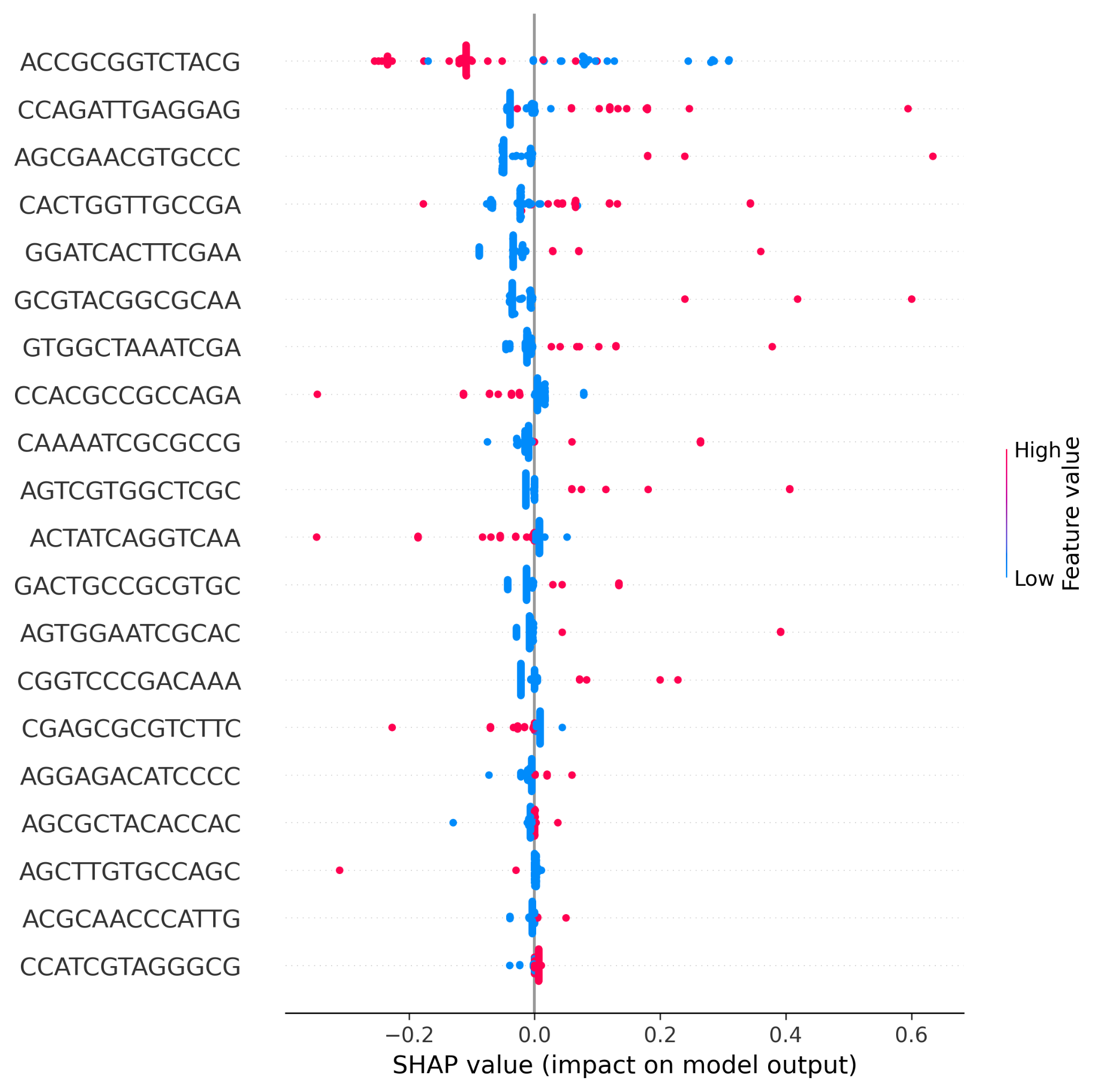


**Cephalosporins**

**Supplementary Fig 2: CEFTAZIDIME**

**
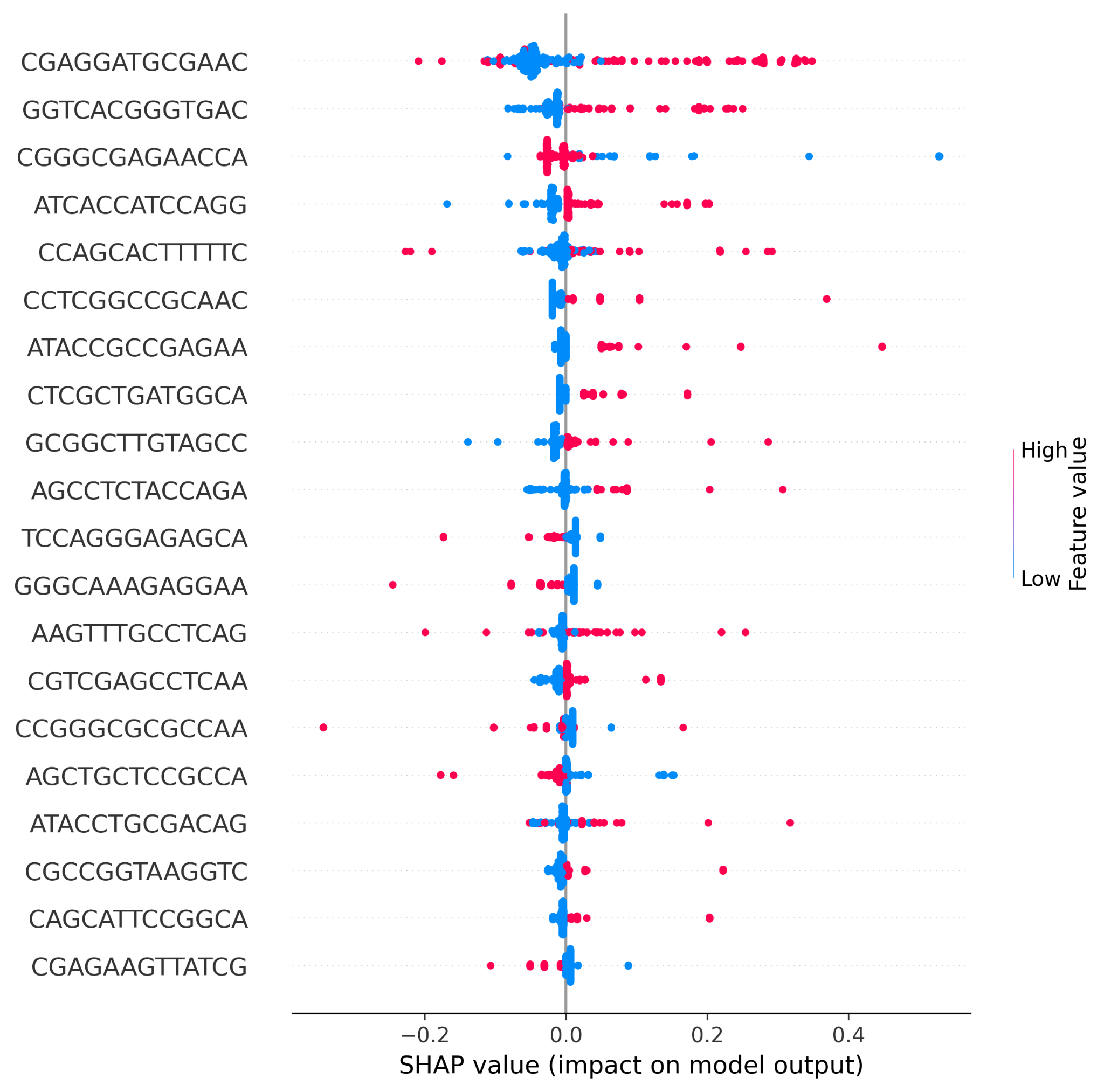
**

**Cephalosporins**

**Supplementary Fig 3: CEFEPIME**

**
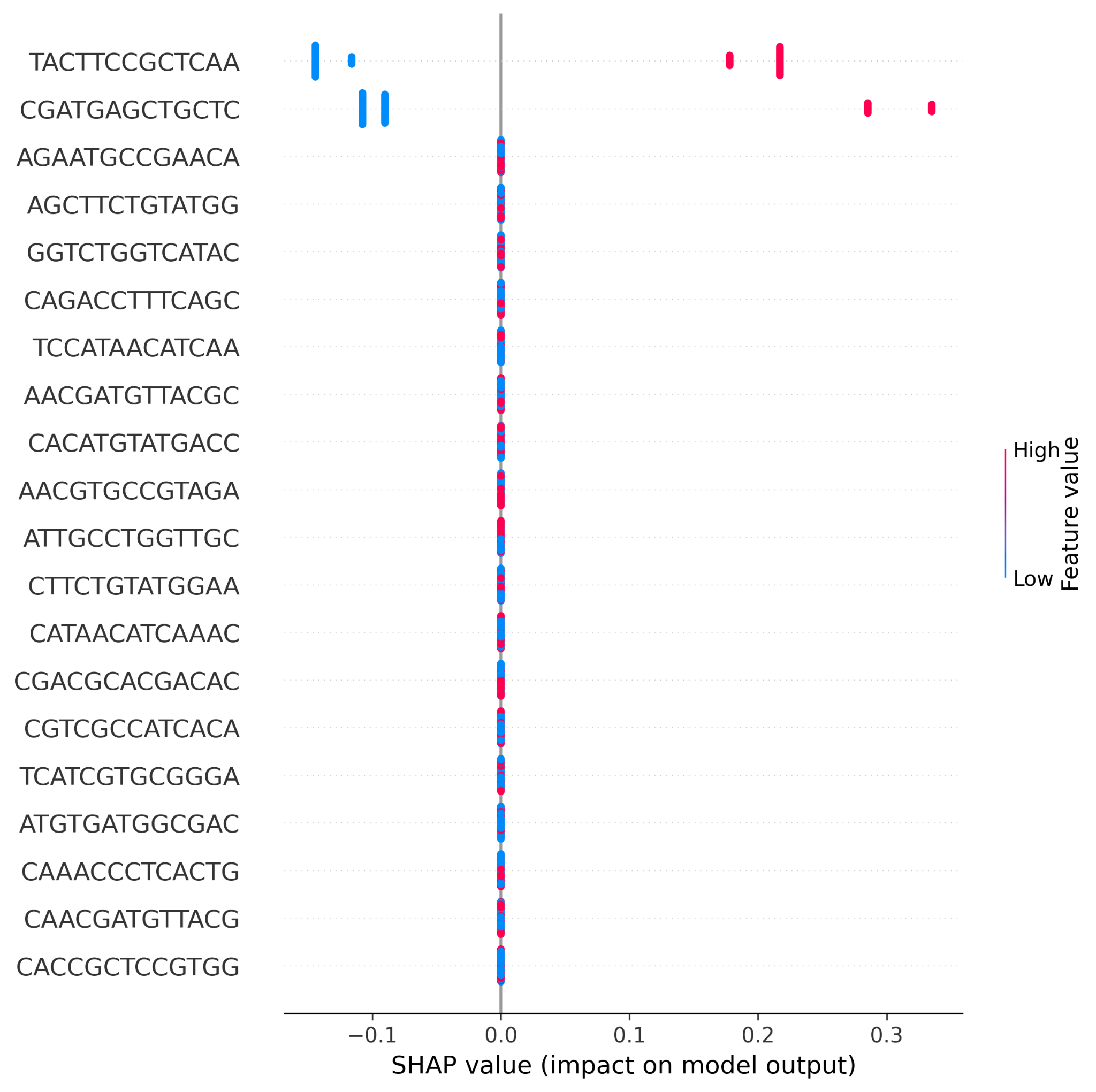
**

**Monobactams**

**Supplementary Fig 4: AZTREONAM**

**
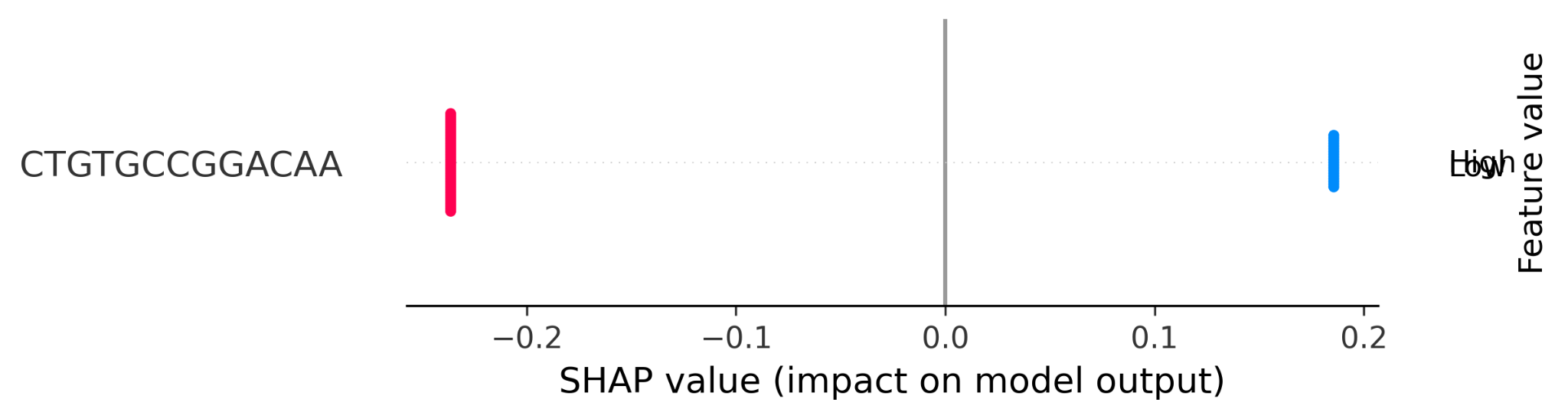
**

**Fluoroquinolones**

**Supplementary Fig 5: CIPROFLOXACIN**

**
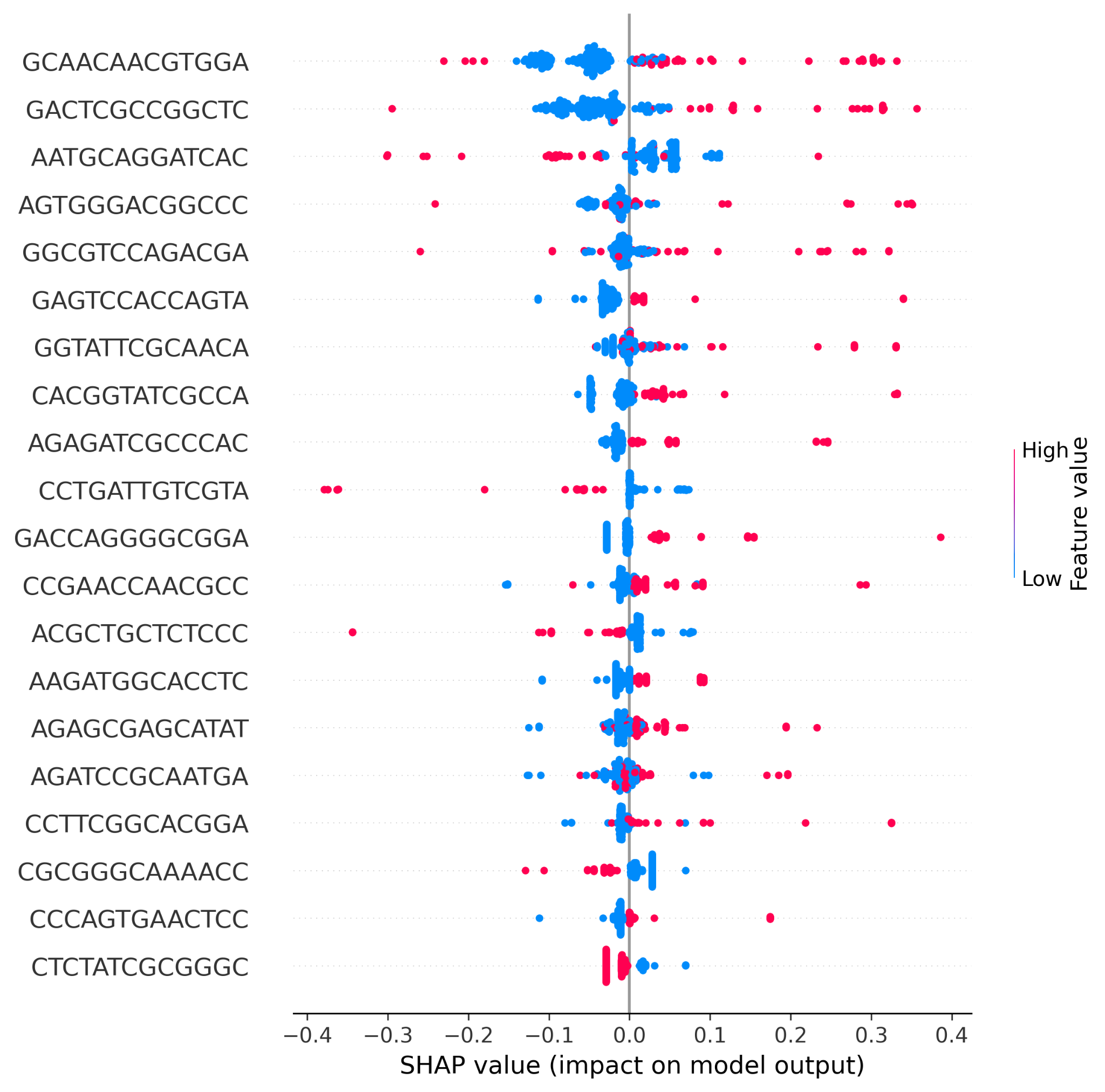
**

**Fluoroquinolones**

**Supplementary Fig 6: LEVOFLOXACIN**

**
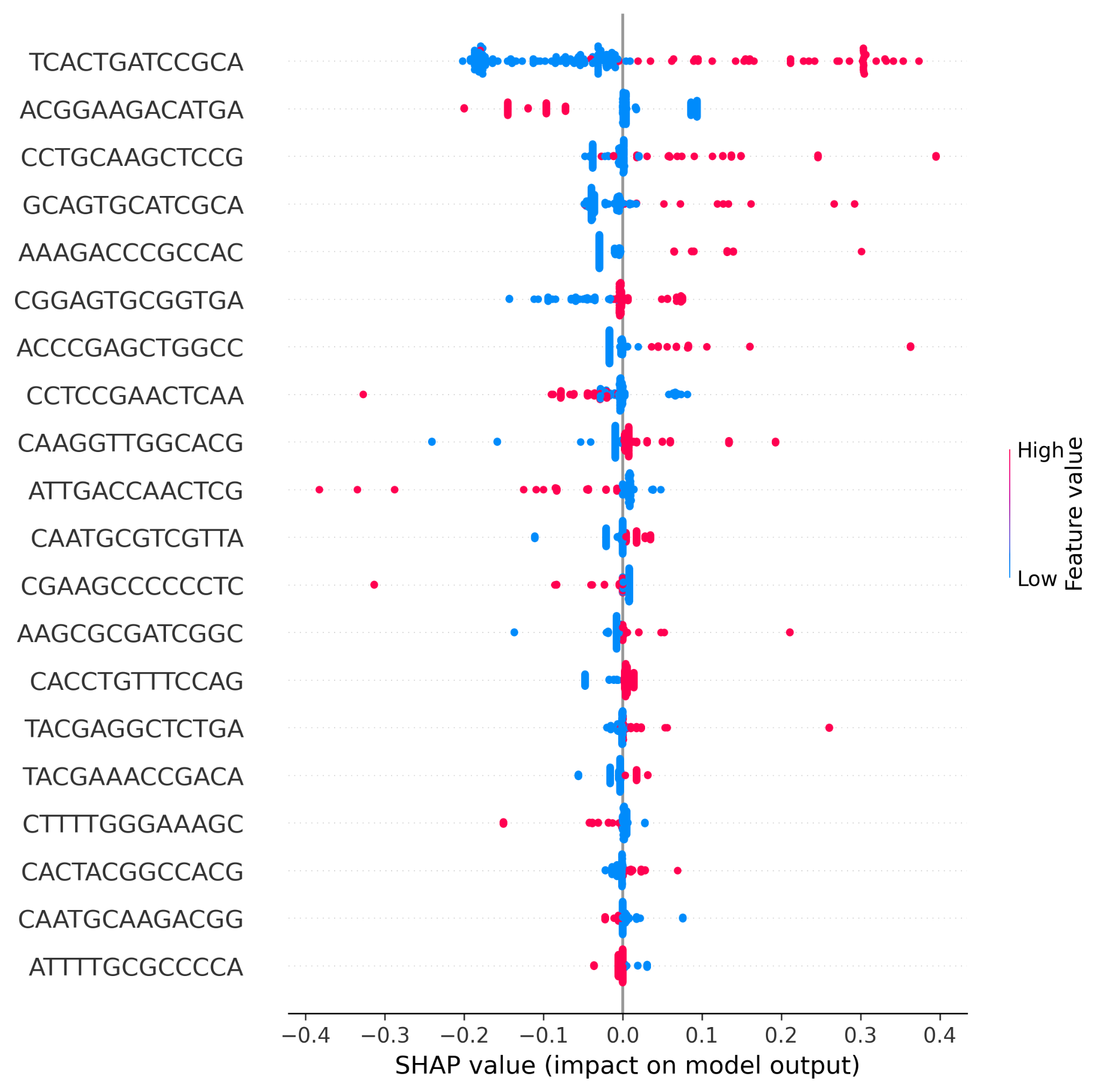
**

**CARBAPENEMS**

**Supplementary Fig 7: DORIPENEM**

**
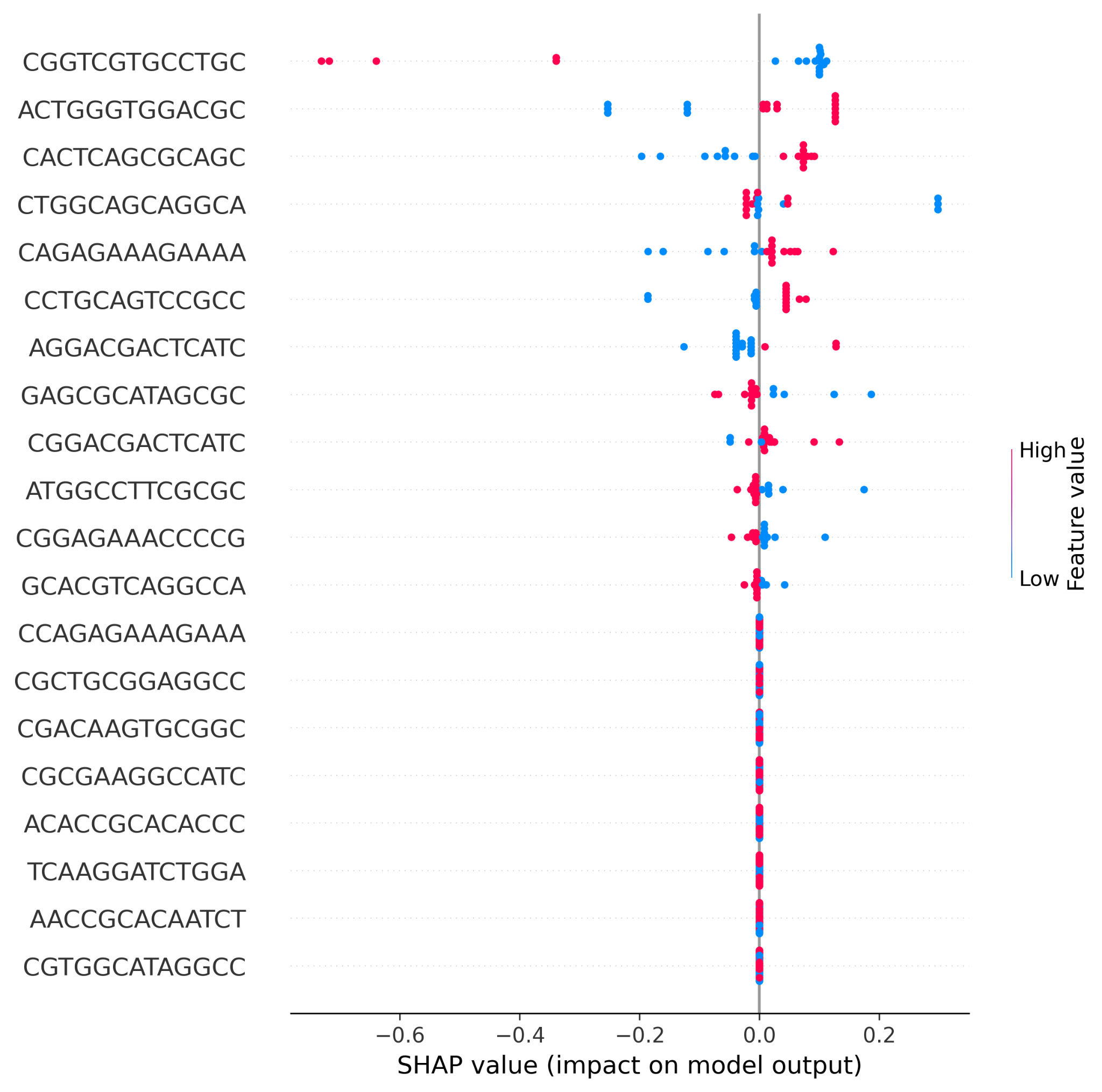
**

**CARBAPENEMS**

**Supplementary Fig 8: IMIPENEM**

**
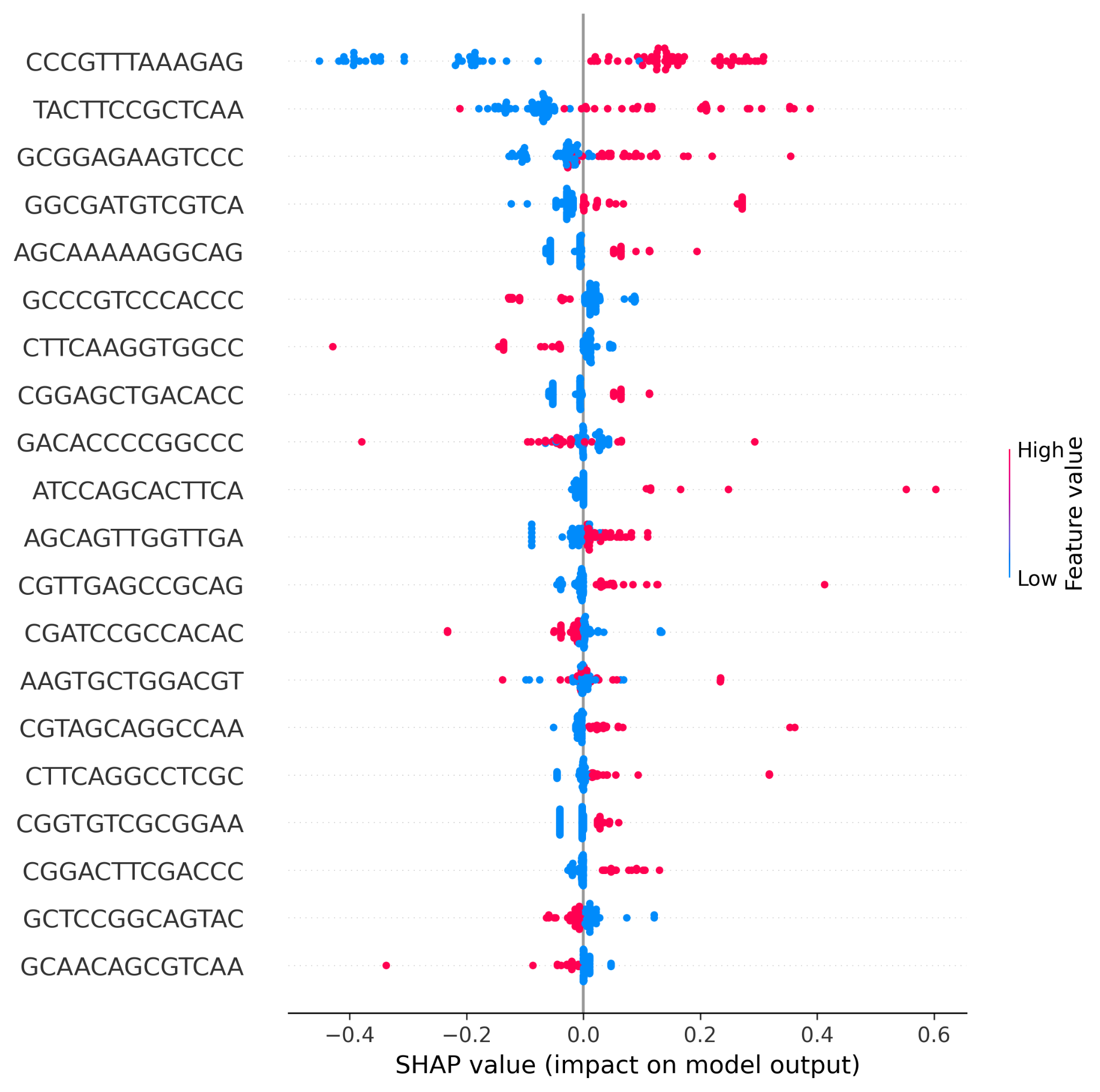
**

**CARBAPENEMS**

**Supplementary Fig 9: MEROPENEM**

**
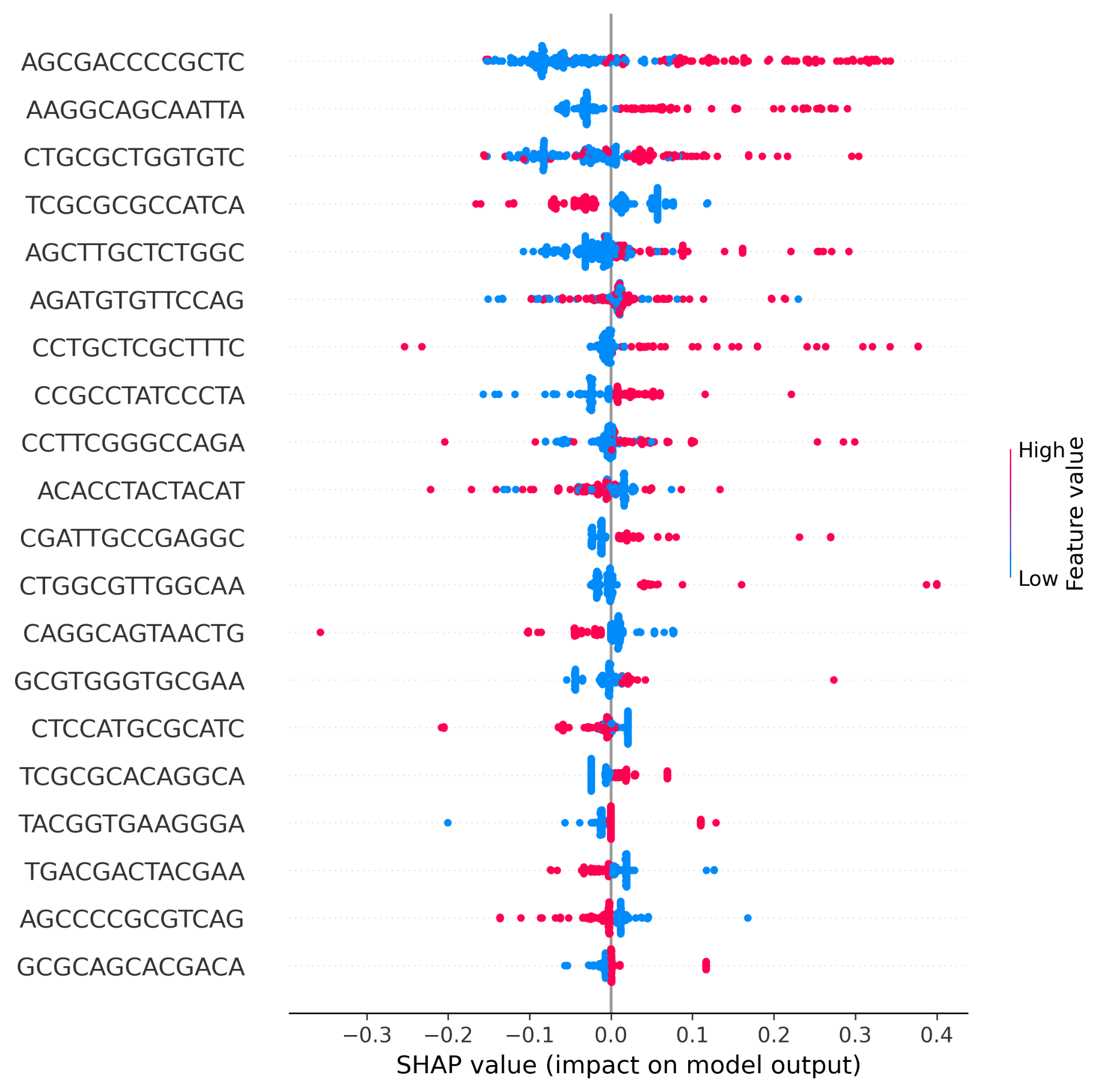
**

**AMINOGLYCOSIDES**

**Supplementary Fig 10 : Amikacin**

**
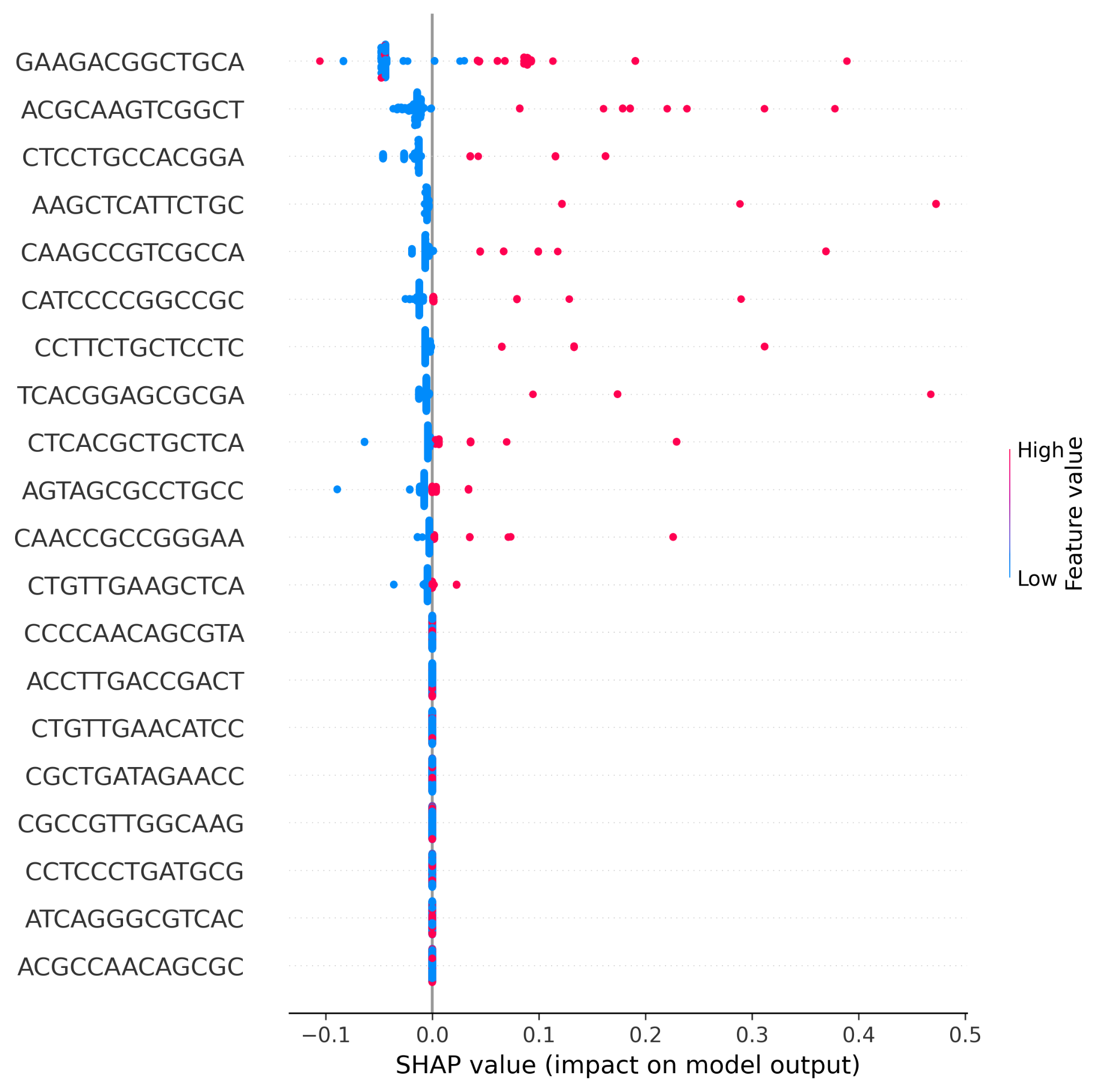
**

**AMINOGLYCOSIDES**

**Supplementary Fig 11: Gentamycin**

**
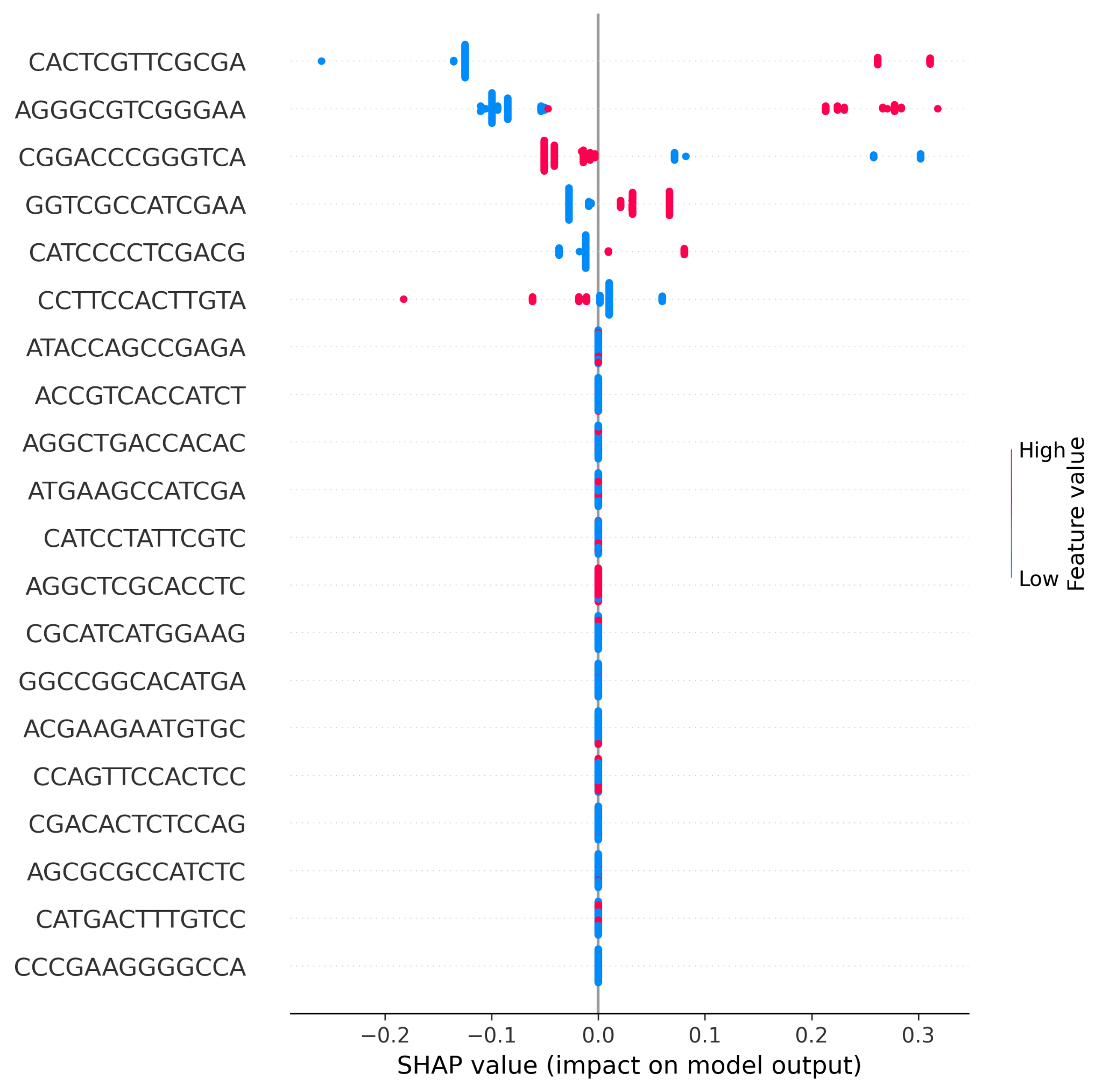
**
